# Investigation on Preparation Process and Storage Stability of Modified Asphalt Binder by Grafting Activated Crumb Rubber

**DOI:** 10.3390/ma12122014

**Published:** 2019-06-23

**Authors:** Juan Xie, Yueming Yang, Songtao Lv, Xinghai Peng, Yongning Zhang

**Affiliations:** 1 School of Traffic and Transportation Engineering, Changsha University of Science and Technology, Changsha 410114, China; 17101030028@stu.csust.edu.cn (Y.Y.); lst@csust.edu.cn (S.L.); pengxinghsi@stu.csust.edu.cn (X.P.); zhangyongning0424@163.com (Y.Z.); 2 National Engineering Laboratory for Highway Maintenance Technology, Changsha University of Science and Technology, Changsha 410114, China

**Keywords:** acrylamide, grafting activated crumb rubber, modified asphalt, orthogonal test, storage stability, softening point difference, segregation index

## Abstract

According to the theory of molecular design, crumb rubber was grafting activated with acrylamide and then used as asphalt binder modifier. An orthogonal three-factor, three-level test was designed to optimize the preparation process of modified asphalt. Softening point, viscosity, rutting factor, ductility, stiffness modulus and creep speed index were selected as evaluation indicators to study the effects of rubber content, shear time and shear temperature by variance analysis and range analysis. The results show that the rubber content had a significant impact on the performance of modified asphalt with grafting-activated crumb rubber, while the shear temperature and shear time had little effect. The grafting activated crumb rubber content of 20%, shear temperature of 170–190 °C, and shear time of 90 min was determined as the reasonable preparation process. Modified asphalt with common crumb rubber (CRMA) and modified asphalt with grafting activated crumb rubber (A-G-R) were prepared, respectively, using the reasonable process to analyze the influence of grafting activation of crumb rubber. The results indicate that A-G-R had smaller softening point difference, lower segregation index and more stable and uniform dispersed phase.

## 1. Introduction

Asphalt pavement has become a main type of pavement structure all over the world because of excellent performance [1,2]. However, with the growth of the traffic volume, neat asphalt binder is difficult to meet modern transport development demands and needs to be modified. Meanwhile, scrap tires called “black pollution” are being generated and accumulated in vast quantities and causing severe environmental issues, so the reclamation of them has become a major concern [3]. Many researchers have shown that scrap tires can be pulverized and added to asphalt to improve the rutting resistance at high temperature, crack resistance at low temperature, aging resistance and fatigue resistance of asphalt pavement, and the environmental pollution can be alleviated at the same time [4,5,6,7]. Therefore, crumb rubber modified asphalt has important economic and environmental benefits and is receiving increasing attention. However, due to the great differences in molecular weight, density, solubility parameters and chemical structure between rubber particle and asphalt, crumb rubber modified asphalt generally has poor thermal storage stability and is easy to segregate [8], which brings inconvenience to the transportation and use of rubber asphalt [9], and also seriously restricts the practical application and rapid development of crumb rubber modified asphalt.

Researchers have done a lot of work to improve the storage stability of crumb rubber modified asphalt and the main methods are generally divided into three kinds: (1) Improving the preparation process. Terminal Blending ((TB) rubberized asphalt is representative of this method, in which finer crumb rubber (above 30 mesh), high mixing temperature, high pressure and high shear rate are used. Different from the swelling mechanism of traditional crumb rubber modified asphalt, the desulfurization and depolymerization of rubber play the main role during the preparation process of TB rubberized asphalt, which endows the asphalt with good storage stability and low viscosity, but damages its elasticity and high temperature properties [10,11,12]. (2) Adding additives. Trans-polyoctenamer rubber (TOR) contains unsaturated bonds, which can react with both sulfur in asphalt and sulfur on the surface of rubber particle to form a network structure, and hence greatly improve the storage stability of modified asphalt [13]. Ghaly added SBS and sulfur to rubber modified asphalt, and found that a chemical bond formed between asphalt and rubber, resulting in the improvement of compatibility between crumb rubber and asphalt [14]. In addition, high-density polyethylene (HDPE), low-density polyethylene (LDPE) [15,16] and hydrogen peroxide/compatibilizer [17] can also be used as additives to increase the storage stability of rubber modified asphalt effectively. (3) Activating the crumb rubber. The crumb rubber can be pretreated appropriately before being used to increase its surface activity, thereby enhancing the interaction between it and asphalt [18]. Shatanawi and co-workers [19] used furfural to activate crumb rubber and found that new bonds occurred when mixed with asphalt, consequently improving the storage stability of the modified asphalt. Liang [20] demonstrated that the specific surface area of crumb rubber was enlarged by microwave radiation, and as was the reaction area between rubber and asphalt. Li [21] selected polyamide possessing good compatibility with crumb rubber and base asphalt to treat crumb rubber by polymer coating method, and obtained modified asphalt with good storage stability. By this way, crumb rubber can be modified purposefully according to the need of application, the mechanism is simple and the effect is obvious.

In this study, to improve the performance of modified asphalt, especially storage stability, the crumb rubber was activated by acrylamide through chemical grafting method based on the theory of molecular design. An orthogonal three-factor, three-level test was designed to optimize the preparation process: the softening point, 175 °C viscosity, 60 °C rutting factor, 5 °C ductility, −24 °C stiffness modulus and creep rate were chosen as evaluation indices to determine reasonable rubber content, shear time and shear temperature. CRMA was also prepared as the reference via the optimized process to study the influence the grafting activation of crumb rubber on the storage stability of modified asphalt.

## 2. Experimental

### 2.1. Materials

Neat asphalt binder of grade 90 provided by Maoming Weilong Petrochemical company (Maoming, China) and crumb rubber with 60–80 mesh obtained from Hengshui Zehao Chemical Company (Hengshui, China) were used, and the performance parameters are shown in Table 1 and Table 2, respectively. Acrylamide and potassium persulfate were of analytical grade, and supplied by Tianjin Hengxing Chemical Reagent Company (Tianjin, China).

### 2.2. Grafting Activation of Crumb Rubber

The crumb rubber was purified with acetone first and then activated. The specific steps are as follows: Firstly, 50 g crumb rubber, 1.25 g potassium persulfate and 20 g acrylamide were added to 500 mL ultra-pure water in the round-bottom flask. Then, the round-bottom flask was put into the magnetic stirrer with water-bath temperature control. The reaction time was 4 h and the reaction temperature was 65 °C. Finally, after washing and drying, the crumb rubber grafted with acrylamide was obtained.

### 2.3. Preparation of A-R-G

There is a relatively complex reaction process between rubber and asphalt in the preparation of crumb rubber modified asphalt. The performance of rubber asphalt is affected by many factors. Considering the influence of crumb rubber content, shear time and shear temperature, orthogonal three-factor, three-level tests were designed to determine the reasonable preparation process of A-G-R. Orthogonal tests of L_9_ (3^3^) are shown in Table 3.

The steps of preparation are as follows: Firstly, the base asphalt was preheated in an oven at 160 °C for 30 min. Then, crumb rubber was stirred into the heated asphalt under the proposed test conditions. Finally, the prepared modified asphalt was placed in the oven at 160 °C for 1 h.

### 2.4. Test Methods

Conventional properties, such as softening point, ductility and viscosity of modified asphalt with grafting activated crumb rubber, were measured in accordance with JTG-2011. A bending beam rheometer produced by Cannon Instrument Company was used to carry out low temperature creep tests. The asphalt binder samples of 125 mm × 12.7 mm × 6.35 mm were prepared and the tests were conducted according to JTG -T0627-2011. The load was 980 mN and lasted for 240 s; the creep stiffness (S) and creep tests were determined at loading time 60 s. The rheological property of modified asphalt was measured according to JTG-T0628-2011 through a dynamic shear rheometer (DSR, MCR 302, Antongpa, Graz, Austria) under strain controlling mode, with temperature swept between 58 and 88 °C at fixed frequencies of 10 rad/s. Principal rheological parameters including complex shear modulus (G*), phase angle (δ) and rutting factor (G*/sinδ) were obtained.

Storage stability of A-G-R and CRMA were evaluated by separation tests according to JTG-T0661-2011. Tubes with modified asphalt were stored in ovens at 163 °C for 24 h, 48 h, 72 h and 96 h and then frozen at −4 °C for 4 h followed by being divided into three sections. The softening point difference and rutting resistance factor separation index of the upper and lower sections were tested.

The distribution of crumb rubber in modified asphalt after separation tests was observed by using a NIKON80-I fluorescence microscope produced by NIKON Company (Tokyo, Japan).

## 3. Results and Discussion

### 3.1. Influence of Different Factors on the High Temperature Performance

Softening point, 175 °C Brookfield viscosity and 60 °C rutting resistance factor were used as indices to evaluate the high temperature performance of A-G-R. Nine samples were tested and the results are listed in Table 4.

**Figure 1:**
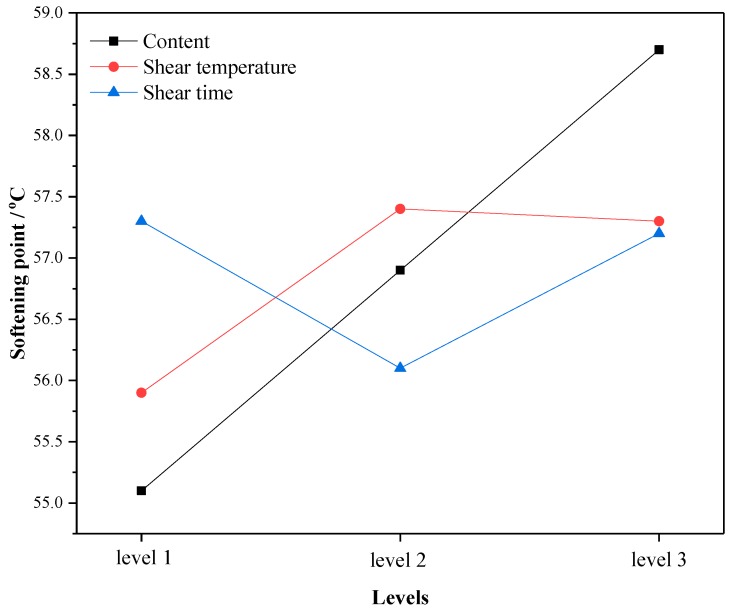
The variation of the mean softening point with the levels.

**Figure 2:**
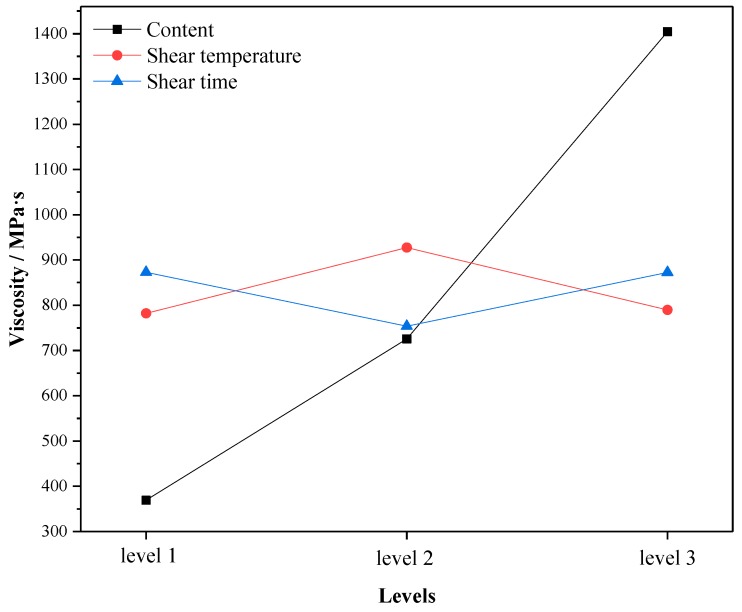
The variation of the mean 175 °C viscosity with the levels.

**Figure 3:**
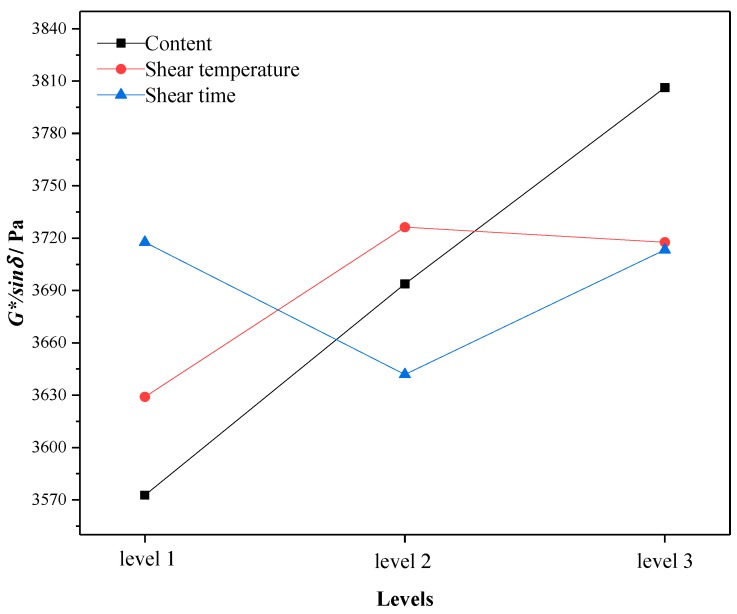
The variation of the mean 60 °C rutting resistance factor with the levels.

**Figure 4:**
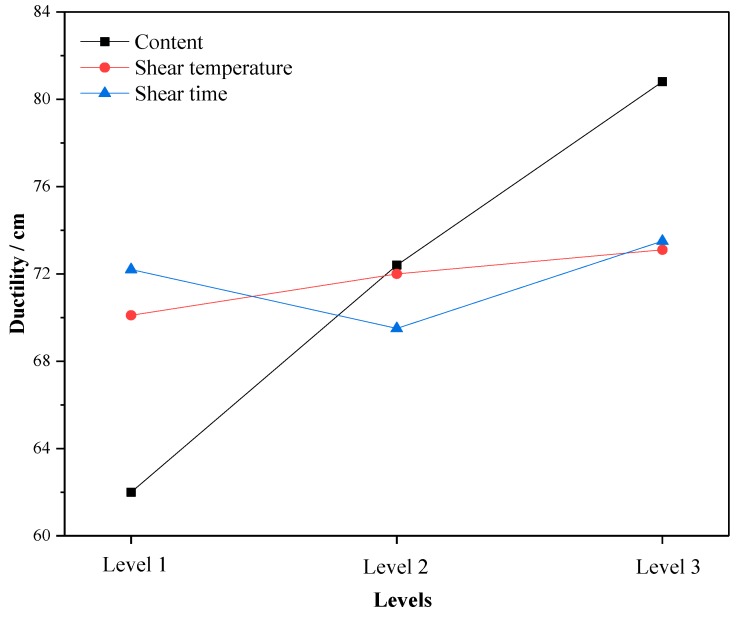
The variation of the mean 5 °C ductility with the levels.

**Figure 5:**
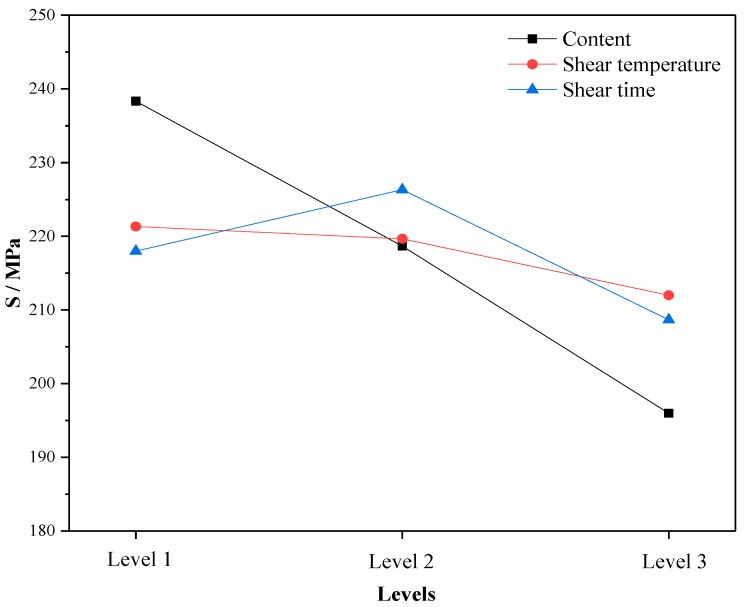
The variation of the mean S with the levels.

**Figure 6:**
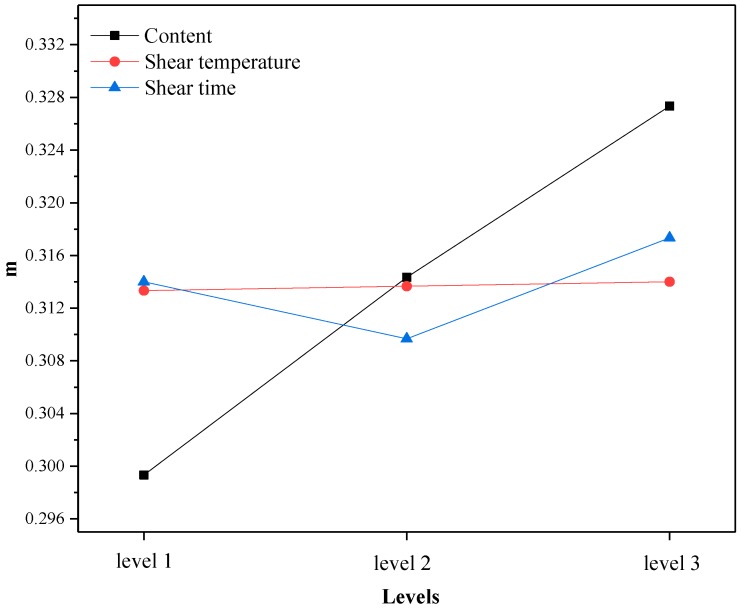
The variation of the mean m with the levels.

Through the average test results of three factors (content, shear temperature and shear time) at the same level in the orthogonal test, the change trend of high temperature performance at different levels was analyzed. The larger the mean value of each test index is, the better the high temperature performance is. The changes of softening point, viscosity and rutting resistance factor with levels under different factors are shown in Figure 1, Figure 2 and Figure 3. Here, Level 1 represents crumb rubber content of 10%, shear temperature of 150–170 °C, and shear time of 30 min; Level 2 represents crumb rubber content of 15%, shear temperature of 170–190 °C, and shear time of 60 min; and Level 3 represents crumb rubber content of 20%, shear temperature of 190–210 °C, and shear time of 90 min. This annotation is also applicable to Figure 4, Figure 5 and Figure 6.

According to Figure 1, Figure 2 and Figure 3, the influence of different factors on the high temperature performance are summarized as follows:

(1) With the increase of crumb rubber content, the softening point and the rutting resistance factor increases linearly with levels. Moreover, the mean value and slope of viscosity increased at the same time. This means that the high temperature performance of A-G-R improved with the increase of crumb rubber content. The main reason is that, with the increase the content, the rubber particles swelled more by absorbing the oil in the asphalt during shearing process, and a more gel-like structure formed, which enhanced the interaction between rubber and asphalt. Besides, the chemical reaction between amide group grafted on rubber polymer chain and the acid groups in asphalt further strengthened the interaction through the formed three-dimensional network structure. The fluidity of asphalt was restricted by the network structure, thus the viscosity and high-temperature deformation resistance of A-G-R was improved.

(2) As shear temperature rose, the high temperature performance of A-G-R increased first and then decreased. This is because the viscosity of the matrix asphalt was lowered due to the increase of the temperature, the crumb rubber was more easily dispersed and the degree of swelling was improved. Thus, the chemical reaction between amide group grafted on rubber molecular chain and acid group in asphalt was more intense and sufficient. However, when the temperature was too high, the aging of the asphalt and the desulfurization reaction of the rubber became more serious, resulting in the decrease in high temperature performance [12].

(3) With the prolonging of shear time, the high temperature performance of A-G-R declined first and then rose. This is might be due to that the light component of asphalt being absorbed by rubber particles, and the swelling being dominant in early stage. The content of light component in asphalt and the swelling decreased gradually with time, which weakened the cross-linking among rubber [22]. However, with prolonging the shear time, the amide group grafted on rubber molecular chain began to react with the acid group in asphalt, which led to the improvement of high temperature performance.

### 3.2. Influence of Different Factors on the Low Temperature Performance

The 5 °C ductility, −24 °C stiffness modulus (S) and creep rate (m) were used as indices to evaluate the low temperature performance of A-G-R. The nine samples shown in Table 4 were tested and the results are shown in Table 5.

As a kind of viscoelastic material, asphalt exhibits obvious brittleness at low temperature. The ductility at 5 °C of asphalt is usually used to identify its tensile properties at low temperature, which can reflect the crack resistance of the binder. However, for modified asphalt, ductility cannot reflect the low temperature mechanical performance completely. Therefore, the S and m obtained from bending beam rheometer (BBR) test were used to characterize the crack resistance and stress relaxation ability of modified asphalt at low temperature. The lower is the S value and the higher is the m value, the better is the low temperature performance of asphalt.

The following results can be seen in Figure 4, Figure 5 and Figure 6:

(1) With the increase of crumb rubber content, the ductility and m increased with levels, and the stiffness modulus S exhibited a downward trend. That is, the low temperature performance of A-G-R improved when the crumb rubber content increased. This is because the increasing rubber particles absorbed more light components of asphalt, which boosted the proportion of the elastic component in the whole system. Besides, as a kind of high elastic material, rubber can withstand large deformation and reduce the hardness of asphalt at low temperature [23], which makes rubber modified asphalt have good ductility and tenacity. The nodes of the three-dimensional network structure formed by the interaction of asphalt and crumb rubber can improve the low temperature relaxation ability of modified asphalt [23]. With the chemical reaction between amide group grafted on rubber molecular chain and acid group in asphalt progress, the nodes of the three-dimensional network structure increased, and the low temperature relaxation ability of A-G-R increased consequently.

(2) As shear temperature rose, the low temperature performance of A-G-R improved. The interaction between rubber and asphalt accounts for this result. It was a key factor in determining the low temperature performance of modified asphalt [24,25], and was strengthened by the swelling of rubber and chemical reaction between rubber and asphalt when temperature rose.

(3) With the prolonging of time, the low temperature performance of A-G-R decreased first and then increased. This is because, after a period of swelling, the decrease of light oil content led to the weakening of the interaction between rubber and asphalt, which affected the low temperature performance of modified asphalt. With the extension of shear time, the amide group grafted on rubber molecular chain began to react with the acid group in asphalt, which strengthened the interaction and network structure between rubber and asphalt, hence the low-temperature performance was significantly improved.

### 3.3. Influence Extent of Different Factors on the Properties of A-G-R

To obtain the weight and significance of each factor on the performance of A-G-R, the range analysis and variance analysis were carried out to determine the influence extent.

The range is defined as the difference between the maximum average value and the minimum average value at each level under the same factor. The range reflects the influence extent of the factor on the performance index, and the larger the range is, the greater the influence extent of this factor is. In Table 6, it can be seen that the order of influence extent of each factor on three high temperature performance indices of A-G-R was as follows: crumb rubber content > shear temperature > shear time. The order of influence extent of each factor on three low temperature performance indices was as follows: crumb rubber content > shear time > shear temperature. Those indicate that the improvement of high and low temperature performance of A-G-R caused by the increase of crumb rubber content was the most obvious. In contrast, shear temperature had a greater influence on the high temperature performance, and shear time had a greater influence on the low temperature performance.

The variance analysis was used to study the influence rule of each factor on the test index and determine whether the influence of each factor was significant. The values of the high and low temperature performance indices were analyzed by single factor ANOVA analysis via SSPS 19.0 statistical analysis software. When the F value is greater than the critical value F_α_ (n_1_,n_2_), if the significance obtained is less than the presupposed significance level α = 0.05, then the factor has a significant impact on the value of the index. Table 7 shows that the rubber content had a significant effect on both the high and low temperature properties of A-G-R, while the influence of shear temperature and shear time was not significant. In particular, the significance of shear temperature on the low temperature performance was close to 1, indicating that the influence extent of shear temperature could be ignored.

By means of the orthogonal experiment, and analysis of range and variance, it can be seen that crumb rubber content was the main factor affecting the high and low temperature performance of A-G-R, thus it should be determined first during optimization. As shown in Figure 1, Figure 2 and Figure 3, the rise of the three high temperature indices in the range of 15–20% was slightly larger than that in the range of 10–15%. Therefore, considering the high temperature performance, the optimum content should be more than 20%. However, the rise of the three low temperature indices in Figure 4, Figure 5 and Figure 6 was slightly smaller than that in 10–15%, thus the optimum dosage should be between 15% and 20% according to the low temperature performance. By comprehensive consideration, the content was determined to be 20%. Shear temperature had little effect on the low temperature performance of asphalt. Therefore, when determining the shear temperature, the effect of shear temperature on high temperature performance should be mainly considered. Figure 1, Figure 2 and Figure 3 show that the modified asphalt had the best high temperature performance at Level 2, thus the shear temperature is recommended to be 170 °C. In regards to shear time, the results in Figure 1, Figure 2, Figure 3, Figure 4, Figure 5 and Figure 6 indicate that the chemical reaction between amide group grafted on rubber and acid group in asphalt had a significant effect after 60 min. However, the rubber might degrade in large quantities if the shear time were too long [22], which would deteriorate the performance of modified asphalt, hence the reasonable shear time is 90 min.

### 3.4. Storage Stability

**Figure 7:**
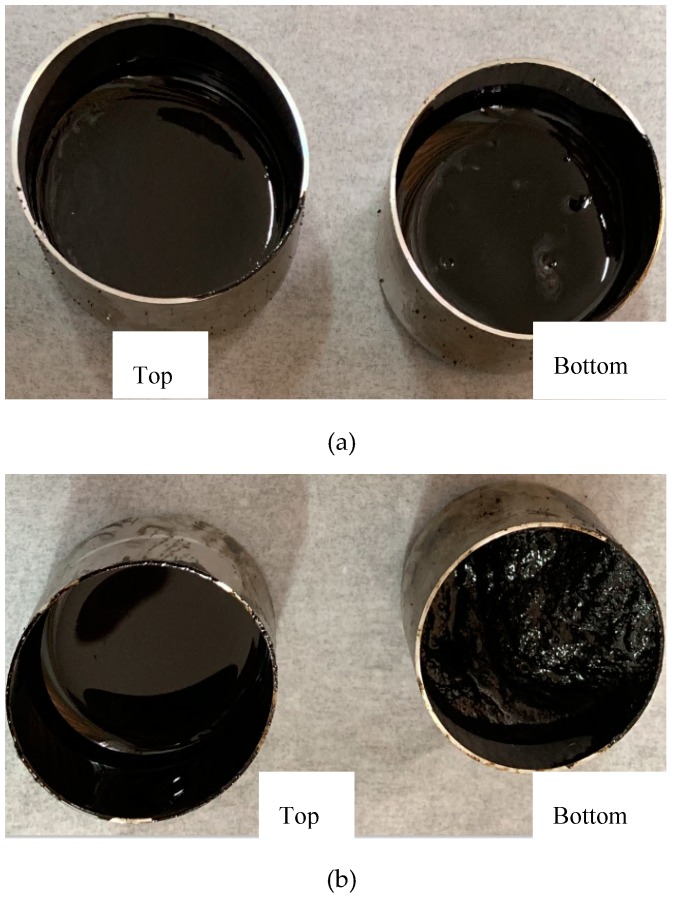
A-G-R (**a**); and CRMA (**b**) stored for 48 h at 163 °C.

Grafted activated crumb rubber modified asphalt (A-G-R) and common crumb rubber modified asphalt (CRMA) were prepared, respectively, through the above optimized process. Segregation tests were carried out on two kinds of modified asphalt. Figure 7 shows the top and bottom of A-G-R and CRMA stored for 48 h at 163 °C. By the naked eye, the phase separation of CRMA was very obvious and much rubber accumulated at the bottom of the aluminum tube. However, the segregation degree of A-G-R was much smaller.

**Figure 8:**
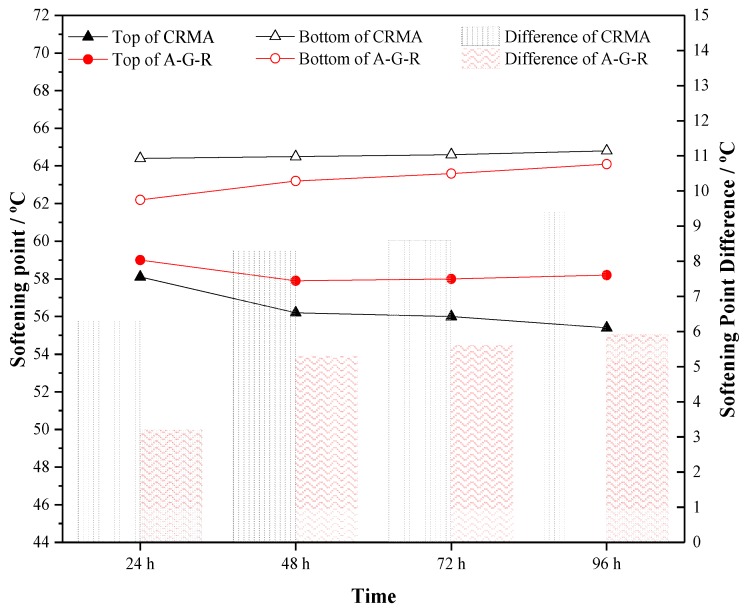
Softening point difference of CRMA and A-G-R.

In addition, the softening points of upper and lower sections were measured and the results are shown in Figure 8, where broken line diagram represents the softening point of asphalt at the top and bottom of tube, and the column diagram represents the difference between them. It can be seen that, for both modified asphalts, the softening points of lower part were higher than that of upper part, and the softening point difference increased with the prolonging of store time, which means that segregation occurred after being stored for a while at high temperature. However, the softening point difference of A-G-R was consistently lower than that of CRMA at all storage times, which means that the A-G-R had better storage stability than CRMA. It still can be explained as the chemical reaction caused by amide groups grafted on rubber and acid groups in asphalt enhanced the interaction and increased the compatibility of the modified asphalt. Furthermore, after being stored for 48 h, the softening point difference of CRMA was still increasing, while that of A-G-R tended to be stable.

A rheological property test has been proven to be an effective way to evaluate the storage stability [26], thus the rutting resistance factor of samples at the top and bottom were measured, respectively. The segregation index (SI) obtained from the temperature sweep tests (from 58 °C to 88 °C) was introduced to estimate the storage stability and compatibility of modified asphalt [27]. SI is defined as the rutting resistance factor ratio of modified asphalt at the bottom and top of tube:

*SI* = (*G*^∗^/*sinδ*) _*bottom*_/(*G*^∗^/*sinδ*) _*top*_ (1)

where (*G*^∗^/*sinδ*) _*bottom*_ and (*G*^∗^/*sinδ*) _*top*_ are the rutting resistance factor of modified asphalt at the bottom and top of tube, respectively. The closer to 1.0 the value of SI is, the smaller the segregation degree of modified asphalt is.

**Figure 9:**
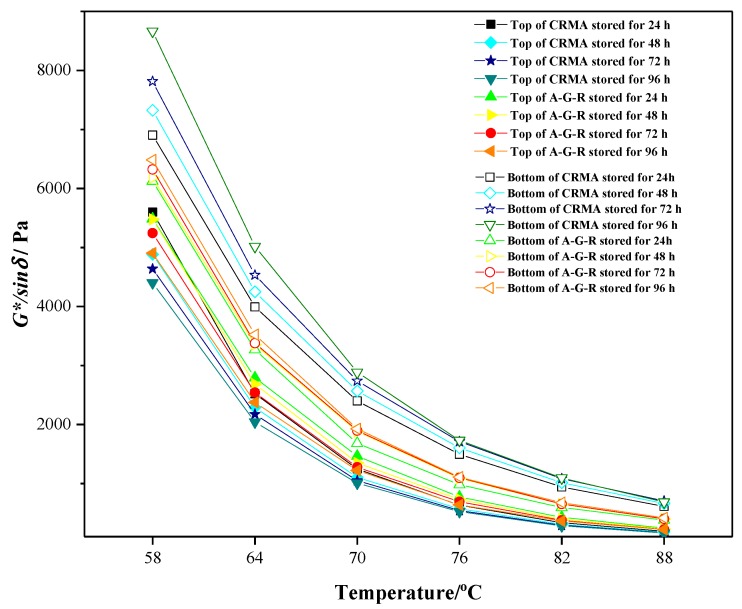
Rutting factor–temperature curve of CRMA and A-G-R.

The results of *G*^∗^/*sinδ* of the upper part and the lower part are shown in Figure 9. It can be seen that, with the increase of temperature, the rutting resistance factor of all samples descended remarkably. The *G*^∗^/*sinδ* of the lower part was higher than that of the upper part, and, with increase of storage time, the *G*^∗^/*sinδ* of the upper part decreased and the *G*^∗^/*sinδ* of the lower part increased. This can be illustrated by that rubber particles with bigger density accumulating at the bottom of tube due to segregation, resulting in higher content of rubber in asphalt at bottom and bringing with better high temperature performance, which is consistent with the results of orthogonal tests. Besides, the *G*^∗^/*sinδ* values of A-G-R were higher than that of CRMA in general, indicating that grafting activated rubber had better modification effect on the high temperature performance than common rubber. Another possible reason is that a chemical reaction occurred between grafting activated rubber and asphalt.

**Figure 10:**
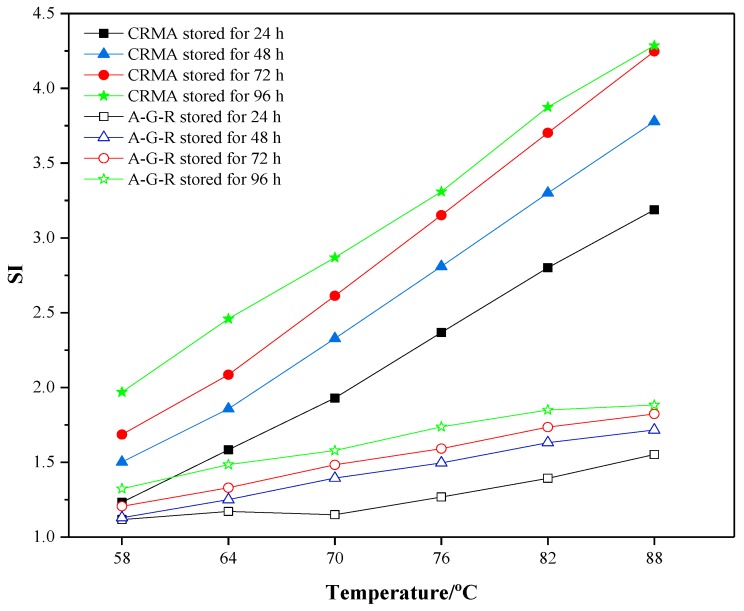
Segregation index curves of CRMA and A-G-R.

To further study the effect of grafting activation of rubber on the storage stability and compatibility of modified asphalt, the SI was calculated from the results of DSR, as shown in Figure 10. It can be found that, for both modified asphalts, the SI values increases with storage time and temperature, indicating the generation of segregation and the decline of the storage stability. However, it is noticeable that the SI values of A-G-R were much smaller than those of CRMA in the whole testing range, and the maximums of A-G-R and CRMA were 1.88 and 4.26, respectively, at 88 °C after being stored for 96 h. Thus, it is believed that the storage stability and compatibility of A-G-R are much better than those of CRMA, which is due to the grafting activation of rubber by acrylamide.

### 3.5. Morphology

**Figure 11:**
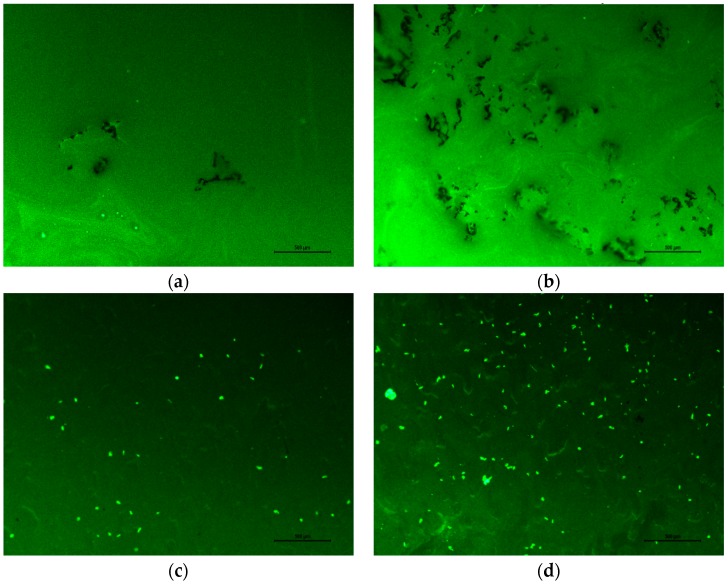
FM images of CRMA and A-G-R stored for 48 h. (**a**) The image of upper CMRA; (**b**) The image of lower CMRA; (**c**) The image of upper A-G-R; (**d**) The image of lower A-G-R.

The Fluorescence Microscope (FM) test was used to explore the morphology of the upper and lower samples of modified asphalt stored for 48 h, especially the dispersion state of crumb rubber in the base asphalt, which reflects the compatibility between modifier and asphalt. In Figure 11a,b, it can be seen that, for CRMA after 48 h of heat storage, there was significant a difference between the upper and lower samples. The rubber sunk due to bigger density than asphalt and agglomerated at the bottom of tube, which led to the reduction of rubber content at the top and aggregation of particles at the bottom. Meanwhile, there was a distinct interface observed between rubber and asphalt, indicating their poor compatibility [28]. Compared with CRMA, there were both physical and chemical reactions in A-G-R, thus the segregation degree in A-G-R was dramatically decreased and the rubber distributed uniformly without agglomeration, regardless of the part shown in Figure 11c,d. Therefore, the analysis of FM images further proved that the storage stability and compatibility of modified asphalts are improved by the grafting activation of rubber with acrylamide.

## 4. Conclusions

Crumb rubber was activated by acrylamide first based on the theory of molecular design and then used as asphalt modifier. To optimize the preparation process of modified asphalt, an orthogonal three-factor, three-level test was designed, and the effects of preparation parameters on the high and low temperature performance were analyzed. Moreover, CRMA and A-G-R were prepared using the optimized process conditions to study the influence of grafting activation of rubber on the storage stability of modified asphalt. The following conclusions can be drawn:

(1) Rubber content is the most significant processing parameter, and the high and low temperature performance of modified asphalt were remarkably improved with it.

(2) With the increase of shear temperature, the high temperature performance increased first and then decreased, while the low temperature performance increased continually.

(3) As shear time increase, the high and low temperature performance of modified asphalt decreased first and then increased.

(4) Combined with the results of orthogonal test and the analysis of range and variance, the optimized preparation technology is recommended as: rubber content of 20%, shear temperature of 170 –190 °C and shear time of 90 min.

(5) After being stored at 163 °C for a while, the softening point difference and SI of A-G-R were much lower than that of CRMA, which means the segregation degree of A-G-R was smaller than that of CRMA.

(6) During the storage period, the dispersion of rubber in A-G-R was more uniform and stable than that of CRMA.

(7) The chemical reaction between the amide groups grafted on the rubber and acid groups in the asphalt enhanced the interaction of the mixture system, thus improving the storage stability and compatibility of modified asphalt.

## Figures and Tables

**Table 1:** Properties of base asphalt binder.

Item	Units	Test Results	Standard
Penetration (25 °C, 100 g, 5s)	0.1 mm	78.1	JTG-T0604-2011
Softening temperature	°C	44.9	JTG -T0606-2011
Ductility (5 °C, 5 cm/min)	cm	>100	JTG-T0605-2011
Kinematic viscosity (135 °C)	Pa s	108.5	JTG-T0625-2011
Density	g/cm^3^	1.009	JTG-T0603-2011
RTFO treated at 163 °C for 85 min			
Quality change	%	−0.063	JTG-T0610-1-2011
Residual penetration ratio (25 °C)	%	57.5	JTG-T0610-2-2011
Residual ductility (5 °C)	cm	8.9	JTG-T0605-2011

**Table 2:** Properties of crumb rubber.

Item	Result	Standard
Water content (%)	0.98	HG/TXXX-2001 7.2.2
Ash content (%)	9.2	GB4498
Acetone extract content (%)	13.5	GB/T3516
Density (g/cm^3^)	0.95	GB/T533
Tensile strength (MPa)	6.5	GB/T528
Elongation at break (%)	860	GB/T52

**Table 3:** Schemes of orthogonal test.

Number	Factors and Levels		
	Content (%)	Shear Temperature (°C)	Shear Time (min)
1	10	150–170	30
2	10	170–190	90
3	10	190–210	60
4	15	150–170	90
5	15	170–190	60
6	15	190–210	30
7	20	150–170	60
8	20	170–190	30
9	20	190–210	90

**Table 4:** Orthogonal test results of high temperature performance index of A-G-R.

Number	1	2	3	4	5	6	7	8	9
Softening point/	54.3	56.0	54.9	56.5	56.5	57.8	57.0	59.8	59.2
175 °C Viscosity/MPa·s	333.3	470.5	303.7	771.6	715.9	689.8	1242.0	1595.6	1375.8
60 °C rutting factor/Pa	3523.0	3633.3	3561.9	3665.7	3665.7	3750.1	3698.2	3879.8	3840.9

**Table 5:** Orthogonal test results of low temperature performance index of A-G-R.

Number	1	2	3	4	5	6	7	8	9
5 °C Ductility/mm	61.8	62.6	61.5	73	71.3	72.9	75.6	82	84.8
S/MPa	246	231	238	210	233	213	208	195	185
m	0.300	0.302	0.296	0.318	0.311	0.314	0.322	0.328	0.332

**Table 6:** Results of range analysis.

Item	Average Value of Range			Significant Sequence of Factor Influence
	Content X	Shear Temperature Y	Shear Time Z	
Softening point/°C	3.6	1.5	1.2	X > Y > Z
175 °C viscosity/MPa·s	1035.3	145	119	X > Y > Z
60 °C rutting factor/Pa	233.6	97.3	75.7	X > Y > Z
5 °C Ductility/mm	18.8	3	4	X > Z > Y
S	42.3	9.3	17.6	X > Z > Y
m	0.028	0.000667	0.007667	X > Z > Y

**Table 7:** Results of variance analysis (α = 0.05).

Factor	Softening Point		175 °C Viscosity		60 °C Rutting Resistance Factor	
	F	Significance	F	Significance	F	Significance
Content	8.383	0.018	59.928	0.000	8.384	0.018
Temperature	0.557	0.600	0.071	0.933	0.557	0.600
Time	0.324	0.735	0.049	0.952	0.324	0.735
**Factor**	**5 °C Ductility**		**S**		**m**	
	**F**	**Significance**	**F**	**Significance**	**F**	**Significance**
Content	34.131	0.001	11.685	0.009	37.596	0.000
Temperature	0.070	0.934	0.138	0.874	0.002	0.998
Time	0.136	0.876	0.482	0.639	0.225	0.805
